# Molecular confirmation of transfusion-transmitted dengue virus infection following platelet transfusion in the Chinese mainland

**DOI:** 10.3389/fcimb.2026.1920581

**Published:** 2026-09-14

**Authors:** Jieting Huang, Ru Xu, Bochao Liu, Qiao Liao, Min Wang, Zhengang Shan, Huishan Zhong, Fenfang Liao, Haojian Liang, Hao Wang, Shijie Li, Yongshui Fu, Huaqin Liang, Xia Rong

**Affiliations:** 1 Institute of Blood Transfusion and Hematology, Guangzhou Blood Center, Guangzhou Medical University, Guangzhou, Guangdong, China; 2 The Key Medical Laboratory of Guangzhou, Guangzhou, Guangdong, China; 3 Department of Hematology, School of Medicine, The Second Affiliated Hospital of South China University of Technology (Guangzhou First People’s Hospital), Guangzhou, China

**Keywords:** asymptomatic infection, blood donor, blood safety, dengue virus, transfusion-transmission infections

## Abstract

**Background:**

Dengue virus (DENV) is considered an emerging threat to blood safety. While transfusion-transmitted DENV (TT-DENV) cases have been documented, TT-DENV has never been reported in Chinese mainland. In this study, a retrospective investigation was conducted after two patients developed dengue fever (DF) following apheresis platelet (PLT) transfusion from the same healthy donor.

**Methods:**

Blood samples from the recipients and the donor were tested for DENV RNA by nucleic acid testing (NAT). Nonstructural protein 1 (NS1) antigen and anti-DENV immunoglobulin M/G (IgM/IgG) antibodies were detected by ELISA. To confirm transmission, DENV E gene fragments were amplified, and whole-genome sequences were obtained by metagenomic next-generation sequencing (mNGS).

**Results:**

Our results revealed that the PLT donor was asymptomatic, tested positive for DENV RNA, and negative for anti-DENV IgM/IgG antibodies at donation. After donation, no dengue-like symptoms was developed. Seroconversion was observed at day 21 postdonation. After receiving the DENV-positive PLT unit, recipient 1 had DF only, while recipient 2 developed DF together with headache, joint/muscle pain, and rash. After transfusion, both recipients were positive for DENV RNA and NS1 while negative for anti-DENV IgM/IgG antibodies. Viral sequences from the donor and recipients were identical and belonged to DENV-2.

**Conclusions:**

To our knowledge, this is the first TT-DENV case reported in the Chinese mainland, revealing the necessity of revising current donor deferral policies and testing strategies that predominantly rely on symptoms and travel history.

## Introduction

Dengue virus (DENV), a member of the genus *Flavivirus*, is classified into four distinct serotypes (DENV-1 to DENV-4), which are endemic in more than 100 countries worldwide and pose an increasing threat to half of the world’s population. The number of cases reported to the World Health Organization has risen dramatically from about 0.5 million in 2000 to 14.6 million in 2024 (World Health Organization, 2025). This number, however, grossly underestimates the true burden, with estimated annual cases ranging from 100 to 400 million each year (World Health Organization, 2025). The regions of the Americas and Asia contributed significant proportions of the global dengue infections due to the favorable geographical and demographical conditions for the breeding of mosquito vectors (species within the *Aedes* genus) (World Health Organization, 2025). In China, dengue fever (DF) is endemic predominantly in southern provinces, particularly Guangdong and Yunnan. They were the epicenters of the 2014 (Guangdong), 2019 (Yunnan), 2023 (both), and 2024 (Guangdong) dengue outbreaks, accounting for over 80% of the national cases (Li et al., 2024). DENV has become a challenge to public health and blood safety in southern provinces of China.

DENV is transmitted in humans primarily through the bite of infected mosquitoes. However, it can also be transmitted via blood components, because around 60%–80% of the infections proceed asymptomatically with viremia (Paz-Bailey et al., 2024). Thus, the impact of DENV on blood safety has become an increasing concern. In 2009, the Association for the Advancement of Blood and Biotherapies (AABB) categorized DENV as a high-priority risk for transfusion-transmitted disease among emerging and reemerging infectious diseases (Stramer et al., 2009). A thorough evaluation of the residual risk of transfusion transmission of DENV (TT-DENV) is therefore essential for the development of preventive strategies. A systematic review summarized an overall DENV viremic rate of 0.2% in blood donors (Gimenez-Richarte et al., 2022a). However, such rates varied substantially, from 0% in nonendemic regions to 14.5% in Colombia during the 2019–2020 outbreak (Ashshi, 2017; Gimenez-Richarte et al., 2022a). Our previous studies also identified DENV viremic blood donors in Guangzhou City, the provincial capital of Guangdong (Liao et al., 2017), implying the potential risk of TT-DENV.

Since the first TT-DENV case was reported in Hong Kong (Chuang et al., 2008), a total of 19 TT-DENV cases have been documented worldwide (Gimenez-Richarte et al., 2022b; Stramer et al., 2012). Critically, the majority of these documented cases occurred during active dengue outbreaks in endemic regions in South America (Levi et al., 2015; Matos et al., 2016; Sabino et al., 2016; Stramer et al., 2012). In the Chinese mainland, however, no TT-DENV case had been reported in DENV-endemic areas or during sporadic outbreaks.

In this study, we report the first confirmed case of TT-DENV in the Chinese mainland: two patients developed dengue fever after receiving apheresis platelets (PLT) transfusion from the same donor who was positive for DENV RNA. The transmission was confirmed by clinical manifestations, serological tests, nucleic acid testing (NAT), and viral sequence analysis. Importantly, this transmission event occurred in early August 2025 when dengue incidence was relatively low, with no major outbreak documented, indicating that DENV poses a threat to blood safety beyond epidemic seasons.

## Materials and methods

### Study subjects

The TT-DENV case involved one blood donor and two recipients: the donor donated 2 units of apheresis PLT on 1 August 2025 (index donation) at Guangzhou Blood Center, Guangzhou, Guangdong, China, which were subsequently transfused to two recipients 2 and 4 days after donation, respectively. Archived blood samples of the donor’s index donation and previous donation (25 days before the index donation) were obtained. A follow-up sample was obtained 21 days after index donation. Pre- (day -1) and posttransfusion (days 10 and 17) blood samples were obtained from recipient 1. For recipient 2, only one posttransfusion sample (day 11) provided adequate volume for all planned tests. Additional blood samples from three individuals who were infected with DENV via mosquito bites were collected for sequence analysis.

### Laboratory tests

DENV nonstructural protein 1 (NS1) antigen, immunoglobulin M (IgM) and G (IgG) antibodies were tested by ELISA (NovaLisa, Gold Standard Diagnostics, Frankfurt am Main, Germany). DENV RNA was tested using the Procleix ArboPlex Assay (Grifols Diagnostic Solutions, San Francisco, CA, USA) on the Procleix Panther System, with a limit of detection (LOD) for DENV-2 of 12.0 copies/mL. Quantification of DENV RNA was performed by real-time PCR assay (Daan Gene, Guangzhou, China), with a LOD of 5.88 × 10^2^ copies/mL and a linear dynamic range from 4 × 10^3^ to 4 × 10^6^ copies/mL. All tests were performed following the manufacturers’ instructions.

### Sequence analysis

Total nucleic acid was extracted from plasma samples using a nucleic acid isolation kit (Daan Gene) on a Smart32 Plus Nucleic Acid Extraction Instrument (Daan Gene), followed by reverse transcription using the PrimeScript RT reagent Kit with gDNA Eraser (Takara Bio, Kusatsu, Japan). DENV E gene fragments (1,592 and 1,685 bp in length for serotypes 1 and 2, respectively) were amplified using serotype-specific primers (Sun et al., 2016) and *Taq* DNA polymerase (Promega, Madison, WI, USA) and sequenced using Sanger sequencing. Whole-genome sequence analysis of DENV was carried out by metagenomic next-generation sequencing (mNGS) methodology. mNGS libraries were prepared directly from the extracted nucleic acid without amplification with DENV-specific primers. The qualified libraries were sequenced on Novaseq (Illumina, San Diego, CA, USA) with 150 bp paired-end (PE150). mNGS reads were cleaned and mapped to NCBI reference sequences for DENV serotypes 1–4 (NC_001477, NC_001474, NC_001475, NC_002640). The mapping reads were then applied to *de novo* assembly to obtain the whole-genome sequence. Sequences of E gene fragments were aligned with NCBI reference sequences for DENV-1 to DENV-4 and other DENV-1 (PV571625, PP563930, OP895916) and DENV-2 (PP411227, OP684216, OQ653841) isolates, followed by phylogenetic tree construction using the neighbor-joining method in MEGA 12.0. Whole-genome sequences were aligned and analyzed for nucleotide/amino acid identity.

## Results

### Characteristics of the donor and recipients

The baseline information of the subjects involved in the TT-DENV case is shown in **Table 1**. The donor was a 28-year-old man. He was required to answer a pre-donation questionnaire specifically designed for dengue/chikungunya fever because of the outbreak of CHIKV in the neighboring city (Foshan, Guangdong). He was a local Guangzhou resident, did not return from a DENV-endemic area, and did not have or develop dengue-like symptoms during the 2 weeks before donation. Recipient 1, a 16-year-old girl with acute lymphoblastic leukemia (ALL), was admitted for headache and severe pneumonia. Blood transfusion was required to treat anemia and thrombocytopenia due to chemotherapy-induced myelosuppression. Three PLT units from three donors and 2 red blood cell (RBC) units from two donors were administered within 5 days. Recipient 2 was a 9-year-old girl suffering from acute monocytic leukemia (AML-M5) and pneumonia. She was admitted for intermittent pain in the left knee without any obvious cause and received PLT transfusion because of thrombocytopenia.

**Table 1:** Baseline information for the donor and recipients in the TT-DENV case.

Subject	Gender	Age	Date of donation/Transfusion	Diagnosis
Donor	Man	28	1 August 2025	–
Recipient 1	Girl	16	3 August 2025	ALL, myelosuppression, severe pneumonia
Recipient 2	Girl	9	5 August 2025	AML-M5, myelosuppression, pneumonia

*ALL*, acute lymphoblastic leukemia; *AML-M5*, acute monocytic leukemia.

### Clinical manifestation and laboratory findings

During this observation, the PLT donor did not show any dengue-like symptoms. However, DENV RNA was identified by NAT at the index donation (4.71 × 10^3^ copies/mL) and during the follow-up (< 4.00 × 10^3^ copies/mL). At the previous blood donation (25 days before the index donation), DENV RNA was not detected. Seroconversion was observed as anti-DENV IgG, and IgM were negative at index donation but positive at follow-up (**Figure 1**, top).

**Figure 1:**
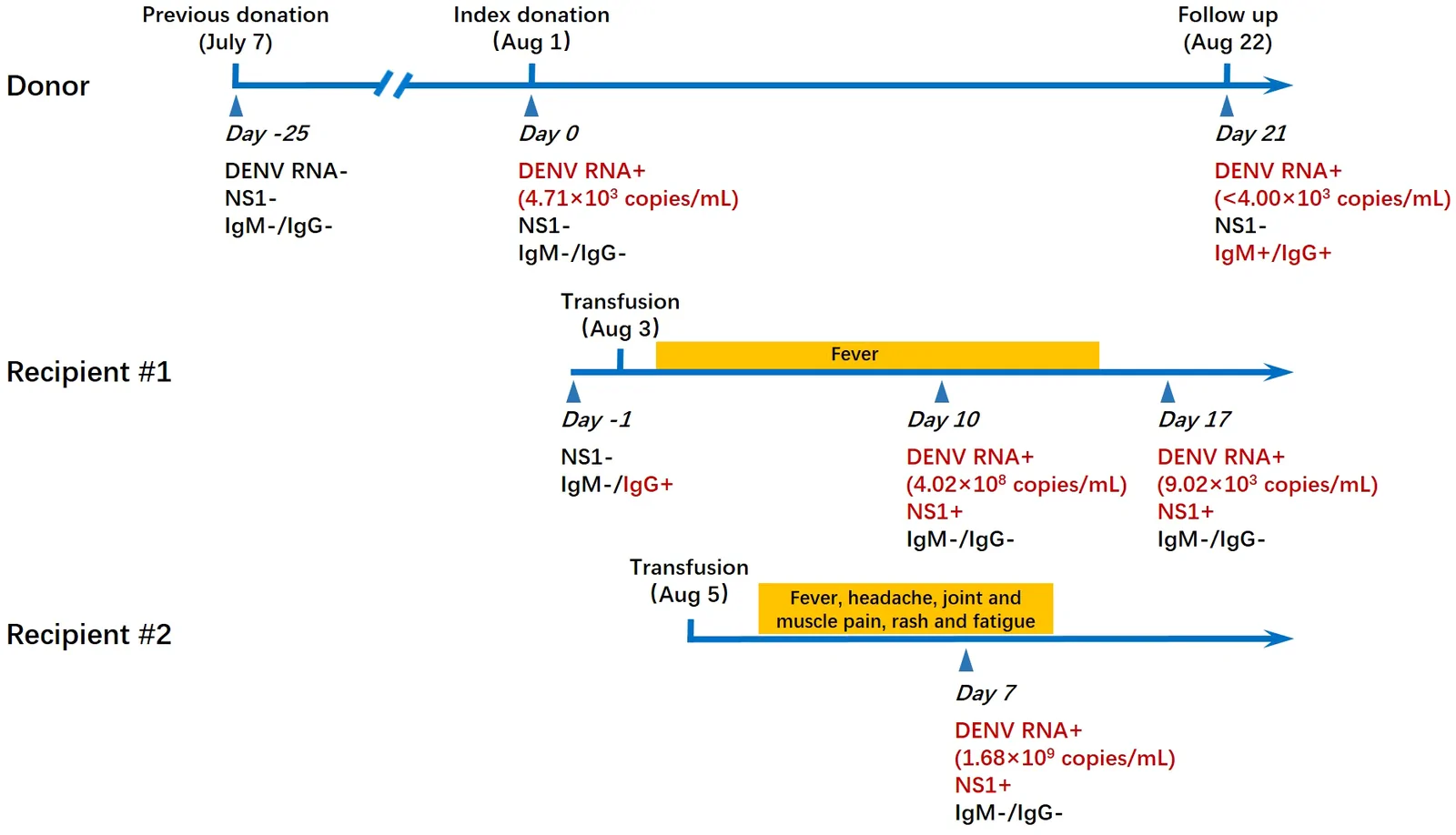
Timeline and laboratory findings of the donor and recipients in the TT-DENV case. Events (blue bars) and laboratory tests (blue triangles) for the donor (top), recipient 1 (middle), and recipient 2 (bottom) are shown. ALL, acute lymphoblastic leukemia; AML-M5, acute monocytic leukemia; DENV, dengue virus; NAT, nucleic acid testing; NS1, nonstructural protein 1; IgM/IgG, immunoglobulin M/G.

Before transfusion with a DENV+ PLT unit, the recipient 1 had fever, but no evidence of acute dengue infection (NS1−/IgM−/IgG+). However, DF was diagnosed 1 day after transfusion (peak temperature 37.8°C). No other dengue-like clinical signs were recorded. The recipient recovered from dengue 14 days posttransfusion. NS1 antigen and a high DENV viral load (4.02 × 10^8^ copies/mL) were detected on day 10 posttransfusion. A 5-log decline of viral load (9.02 × 10^3^ copies/mL) was observed on day 17 posttransfusion, when NS1 antigen remained detectable (**Figure 1**, middle).

Similarly, recipient 2 did not have any dengue-like symptoms before PLT transfusion. DF was diagnosed two days after transfusion, when she developed fever (peak temperature: 39.5°C) that lasted for 8 days, together with headache, joint and muscle pain, rash and fatigue. Supportive therapy was given, and she recovered 10 days posttransfusion. Laboratory tests on the sample obtained 11 days posttransfusion identified the presence of DENV RNA (1.68 × 10^9^ copies/mL) and NS1 antigen (**Figure 1**, bottom). Neither recipient showed seroconversion during the observation period, probably due to their immunocompromised status.

### Viral sequencing and phylogenetic analysis

To confirm the possibility of transfusion-transmission indicated by clinical observation and serological tests, DENV sequences from the donor and recipients were analyzed. E gene fragments from the donor and the two recipients were identical (**Supplementary Figure 1**). They clustered with but were distinguished from the DENV-2 reference sequence and other isolates (**Figure 2**). As controls, three samples from mosquito-borne DF individuals were simultaneously sequenced. These three sequences clustered with DENV-1, and the identities between them were 99.6%–99.7% (**Figure 2**; **Supplementary Figure 1**). Furthermore, whole-genome nucleotide sequences of DENV from the two recipients were obtained by mNGS (**Table 2**). Their sequence identity was over 99.99%, with only one single nucleotide polymorphism at position 3,941 (T>C relative to the DENV reference sequence NC_001474.2), resulting in a non-synonymous substitution of valine (NC_001474.2 and recipient 2) to alanine (recipient 1) at residue 155 in the NS2A protein of DENV (**Figure 3**). mNGS was also performed on the donor’s sample, but only 41 DENV reads were obtained out of 25 million raw reads, which were too few to assemble the whole genome (**Table 2**). The discrepant mNGS results between the recipients and the donor were probably due to their 5-log difference in DENV viral loads. Nevertheless, our results clearly confirmed that two recipients developed dengue after receiving PLT units from the same DENV-positive blood donor.

**Figure 2:**
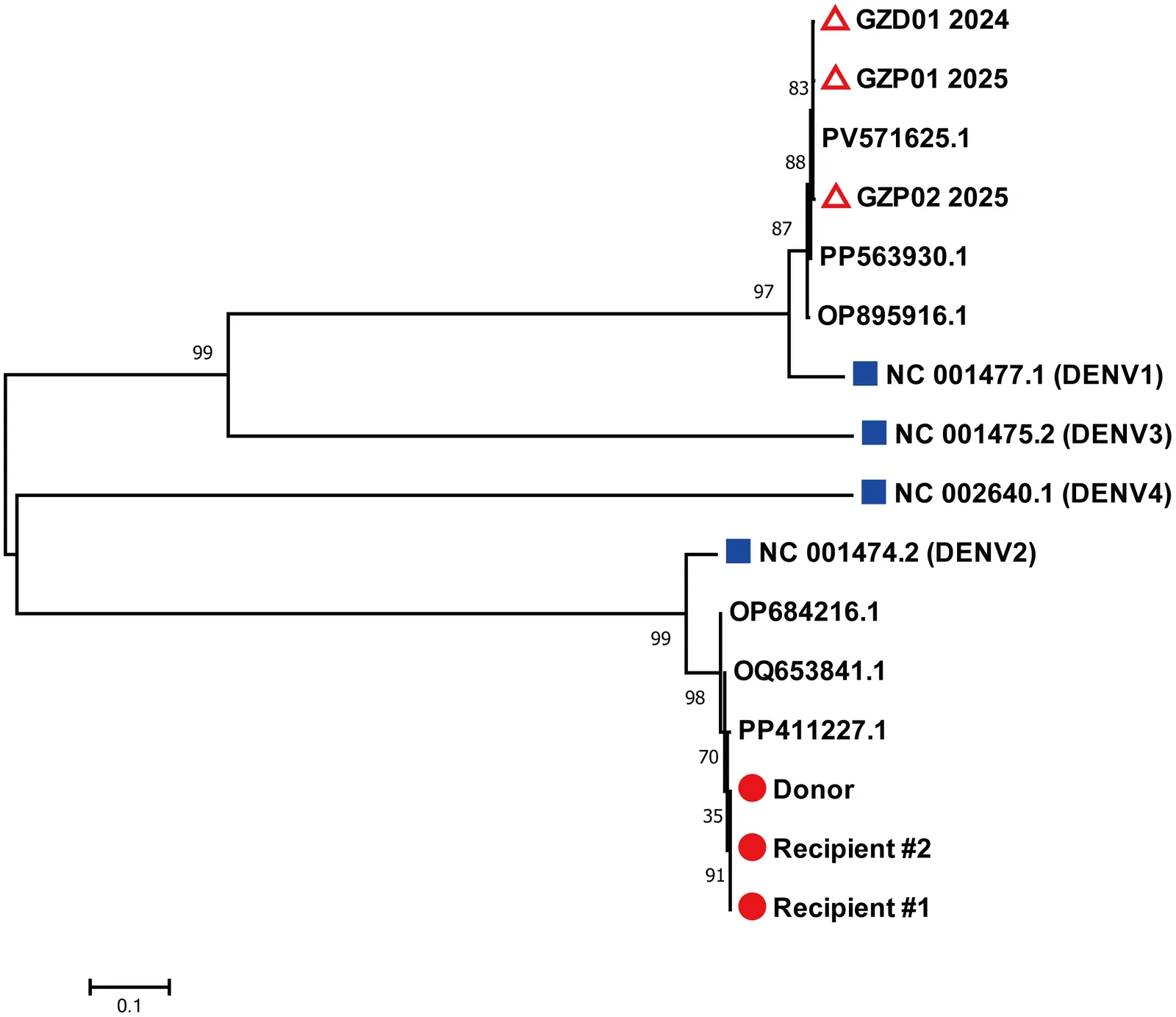
Phylogenetic analysis of the E gene fragment of DENV. A phylogenetic tree of the DENV E gene fragment was constructed using sequences from the donor/recipient (red solid circle), NCBI reference sequences for DENV-1 to DENV-4 (blue square), mosquito-borne DENV controls (red hollow triangle), and isolates of DENV-1 and DENV-2. The percentage of replicate trees in which the associated taxa clustered together in the bootstrap test (500 replicates) is shown next to the branches. The evolutionary distances were computed using the maximum composite likelihood method.

**Table 2:** mNGS results for DENV whole genome assembly.

Sample	Raw reads	Clean reads	DENV reads	Length (bp)	Coverage (%)	Average depth
Donor	25,799,082	25,742,050	41	–^a^	–	
Recipient 1	14,648,376	14,630,164	6,997,249	10,712	100	193,665
Recipient 2	18,822,958	18,710,518	6,456,326	10,710	100	177,636

^a^ Only 41 DENV reads were identified, which were insufficient to assemble the DENV whole genome.

**Figure 3:**
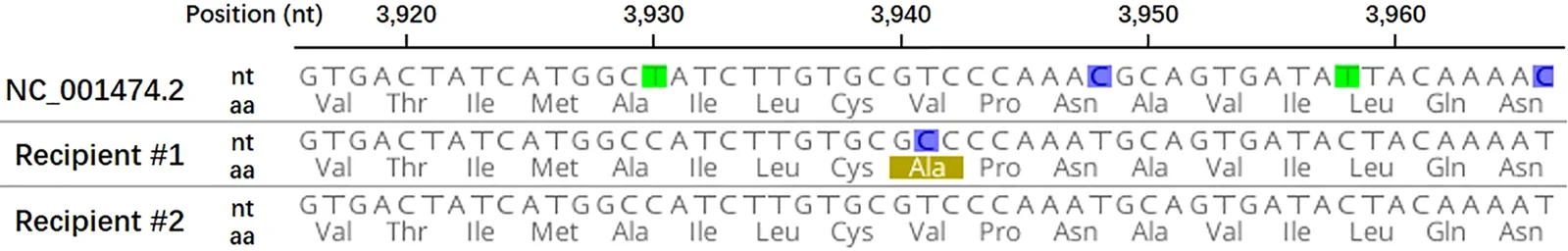
Differences in nucleotide and amino acid sequences of the DENV whole genome between the two recipients. Whole-genome sequences of DENV from recipients 1 and 2 were obtained by mNGS and aligned with the DENV-2 reference strain (NC_001474.2). A fragment of 3,916–3,966 nucleotides (nt) is shown, and polymorphisms at the nt and amino acid (aa) levels are highlighted.

## Discussion

This study reports the first confirmed case of TT-DENV in the Chinese mainland, where one asymptomatic donor infected two recipients through PLT transfusion. Our investigation provided robust evidence for TT-DENV. Two recipients developed fever soon after transfusion, with recipient 2 experiencing particularly significant symptoms. The clinical manifestation pointed to DF, which was further diagnosed by the presence of DENV RNA and NS1 antigen. Since both recipients had been confined to a closed hospital environment for at least 10 days before transfusion, rendering the probability of mosquito exposure exceedingly low, vector-borne transmission of dengue was considered highly unlikely. In addition, both recipients received PLT units from the same donor; thus, transfusion-transmitted infection was suspected. Retrospective investigation indicated that the donor was viremic but seronegative (DENV RNA+/NS1−/IgM−/IgG−) at the time of donation, indicating the infectivity of DENV in the preseroconversion window period. Further evidence confirming TT-DENV was the identical viral E gene fragment sequences among the donor and recipients. In fact, the whole-genome sequences of the two recipients differed by only one nucleotide. Previous studies showed high identity (99.7%–99.9%) of whole-genome sequences among individuals with mosquito-borne DENV in China (Guan et al., 2021; Li et al., 2023; Ma et al., 2025). In this study, high identity (> 99.99%) of the whole genome was also found, suggesting a common origin of DENV in the two recipients.

Compared with other TT-DENV case reports, several aspects of our findings are noteworthy. First, the donor remained entirely asymptomatic, similar to the case described by Levi et al. (2015) in Brazil. This highlights the primary challenge in safeguarding the blood supply: the inability to identify asymptomatic, viremic donors through questionnaire-based screening alone. Second, to our surprise, the viral loads of the recipients were 5-log higher than that of the donor (10^8^ vs.10^3^ copies/mL). Such high viral loads may be attributed to the recipients’ immunocompromised status (chemotherapy-induced myelosuppression), which impaired viral clearance and allowed viral replication. In addition, the viral loads of infused DENV-positive blood products were not associated with the development of TT-DENV. Sabino et al. (2016) found comparable median viral loads between transmission and nontransmission groups, while the infectious dose by blood transfusion could be as low as 36 copies/mL, which was lower than the normal detection limits of real-time RT-PCR. Therefore, although the viral load of our donor appeared to be lower than that of DENV viremic donors in other studies (e.g., > 1 × 10^5^ copies/mL) (Sabino et al., 2016; Stramer et al., 2012), a dose of 1.18–1.42 × 10^6^ copies (the volume of the apheresis PLT unit was 250–300 mL) was able to establish transfusion transmission in both recipients.

The presence of DENV IgG/IgM antibodies seemed to be affected by the route of transmission and the host immune status. In this study, seroconversion of DENV antibodies was observed on day 21 in the donor who was relatively healthy and probably infected by the bite of a mosquito. This was in accordance with the well-established knowledge that IgM and IgG antibodies are detectable from days 4 and 7, respectively, to approximately 12 weeks (Paz-Bailey et al., 2024). In contrast, neither of the recipients had detectable IgG/IgM antibodies at days 17 and 11 posttransfusion, respectively. The absence or delay of antibodies was probably due to the recipients’ immunocompromised status. Notably, recipient 1 was DENV-IgG positive before transfusion, suggesting a prior DENV exposure, yet serial tests at days 10 and 17 posttransfusion were IgG-negative. We speculate that this serological reversal—pretransfusion IgG positivity followed by posttransfusion negativity—reflects the combined effect of two concurrent processes. First, the preexisting IgG may have been depleted through immune complex formation with the high level of viral antigen introduced by transfusion, as circulating viral antigen and preexisting IgG antibody are known to form complexes during secondary DENV infection (Muller et al., 2025). Second, and more critically, under immunocompetent conditions, secondary DENV infection typically elicits a rapid and robust humoral immune response that results in rising antibody levels. However, recipient 1’s immunocompromised status probably impaired memory B-cell reactivation and plasma cell differentiation. The depletion of preexisting antibody followed by the failure of antibody production resulted in negative IgG antibody test results posttransfusion. This interpretation is partially supported by a previously reported TT-DENV case, in which a lymphoma recipient of DENV-positive platelets did not seroconvert, and an aplastic anemia recipient mounted only an IgM response without documented IgG seroconversion during the observation period (Levi et al., 2015). Unfortunately, the pretransfusion sample volume was insufficient for confirmatory testing (e.g., IgG avidity or neutralization assays), and the donor’s DENV serotype could not be determined to assess whether this represented a homotypic or heterotypic secondary infection.

Guangdong province and Yunnan province, which have warm and rainy climates that favored the breeding of mosquitoes, reported over 80% of the national dengue cases (Li et al., 2024). In these two regions, asymptomatic DENV viremic blood donors have been reported (Liao et al., 2017; Yan et al., 2025), revealing the risk of TT-DENV. Deferral of prospective blood donors returning from dengue-endemic regions or those who had dengue-like symptoms was a well-established safety measure to prevent TT-DENV (European Centre for Disease Prevention and Control, 2025). However, the identification of local asymptomatic dengue is difficult, as presented in this study. The DENV-positive donor was overlooked even though he had answered a questionnaire concerning dengue and chikungunya fever. To overcome this limitation, screening of DENV by NAT in all blood products should be considered. This method is highly sensitive, allowing the detection of very low levels of DENV RNA (5 copies/mL) during the preseroconversion window period (Linnen et al., 2008), far exceeding the sensitivity of antigen tests (such as NS1 ELISA), particularly in the early stages of infection (Matos et al., 2016). Lastly, the effectiveness of pathogen reduction technologies for DENV remains inconclusive. Although some studies have demonstrated that pathogen reduction could disinfect DENV in plasma and PLT products (Fryk et al., 2016; Stramer et al., 2022), this approach, however, is not widely implemented in China.

While all blood components have been implicated in TT-DENV, transmission via PLT transfusion appeared to be more frequent in DENV cases than in other arboviruses, such as West Nile virus (Gimenez-Richarte et al., 2022b). PLTs contain host cellular cytosolic organelles that support the translation of virus-encoded proteins (Schubert et al., 2014). *In vitro* studies have demonstrated that DENV can directly bind and replicate in PLT (Simon et al., 2015), probably through the binding of the virus to glycoprotein Ib (CD42b) or other cellular components of PLT (Attatippaholkun et al., 2018). Importantly, the infectivity of DENV in PLT, though it decayed logarithmically, could persist throughout storage (Sutherland et al., 2016). Based on this viral kinetics, around one-tenth of the infectious DENV remained when the PLT unit stored for 3–5 days was transfused to the recipients, which was still capable of establishing TT-DENV in immunocompromised conditions.

Although the TT-DENV in this case was confirmed by clinical manifestation, laboratory tests, and the identity of the viral sequences, there remain some limitations in this study. First, DENV RNA was not tested in the pretransfusion samples of the recipients because of the insufficient volumes. Second, mNGS performed on the donor’s plasma failed to yield sufficient DENV reads for genome assembly, probably due to the relatively low viral load (4.71 × 10^3^ copies/mL), which was ≥ 5 log lower than that of the recipients (10^8^~10^9^ copies/mL).

## Conclusions

This study reports the first confirmed case of TT-DENV in the Chinese mainland. Our findings demonstrate that TT-DENV can occur despite a low viral load (4.71 × 10^3^ copies/mL). The risk of DENV transmission and the threat to blood safety underscore the need for a vigilance system, deferral strategy, and screening methodologies. Highly sensitive screening measures, such as NAT, should be prioritized in endemic regions to mitigate this risk and protect the blood supply, even in nonepidemic settings.

## Data Availability

The original contributions presented in the study are included in the article/**Supplementary Material**, further inquiries can be directed to the corresponding authors.

## References

[B1] Ashshi A. M. (2017). The prevalence of dengue virus serotypes in asymptomatic blood donors reveals the emergence of serotype 4 in Saudi Arabia. Virol. J. 14, 107. doi: 10.1186/s12985-017-0768-7 28599678 PMC5466713

[B2] Attatippaholkun N. Kosaisawe N. Y U. P. Supraditaporn P. Lorthongpanich C. Pattanapanyasat K. . (2018). Selective tropism of dengue virus for human glycoprotein Ib. Sci. Rep. 8, 2688. doi: 10.1038/s41598-018-20914-z 29426910 PMC5807543

[B3] Chuang V. Wong T. Y. Leung Y. H. Ma E. Law Y. L. Tsang O. . (2008). Review of dengue fever cases in Hong Kong during 1998 to 2005. Hong Kong Med. J. 14, 170–177. 18525084

[B4] European Centre for Disease Prevention and Control (2025). Deferral Criteria and Testing Strategies for Dengue Virus in Blood Donors Returning From Affected Areas – Survey Results (Stockholm: ECDC).

[B5] Fryk J. J. Marks D. C. Hobson-Peters J. Prow N. A. Watterson D. Hall R. A. . (2016). Dengue and chikungunya viruses in plasma are effectively inactivated after treatment with methylene blue and visible light. Transfusion 56, 2278–2285. doi: 10.1111/trf.13729 27456861

[B6] Gimenez-Richarte A. De Salazar M. O. Arbona C. Gimenez-Richarte M. P. Collado M. Fernandez P. L. . (2022a). Prevalence of chikungunya, dengue and zika viruses in blood donors: a systematic literature review and meta-analysis. Blood Transfus. 20, 267–280. doi: 10.2450/2021.0106-21 PMC9256504 34694219

[B7] Gimenez-Richarte A. De Salazar M. O. Gimenez-Richarte M. P. Collado M. Fernandez P. L. Clavijo C. . (2022b). Transfusion-transmitted arboviruses: update and systematic review. PloS Negl.Trop. Dis. 16, e0010843. doi: 10.1371/journal.pntd.0010843 36201547 PMC9578600

[B8] Guan J. Li Z. Chen J. Guo Q. Rao Q. Duan S. . (2021). Complete genome characterization of the 2018 dengue outbreak in Hunan, an inland province in central South China. Virus Res. 297, 198358. doi: 10.1016/j.virusres.2021.198358 33667623

[B9] Levi J. E. Nishiya A. Felix A. C. Salles N. A. Sampaio L. R. Hangai F. . (2015). Real-time symptomatic case of transfusion-transmitted dengue. Transfusion 55, 961–964. doi: 10.1111/trf.12944 25605570

[B10] Li Z. Huang X. Li A. Du S. He G. Li J. (2024). Epidemiological characteristics of dengue fever - China 2005-2023. China CDC Wkly 6, 1045–1048. doi: 10.46234/ccdcw2024.217 39502397 PMC11532530

[B11] Li D. Long M. Li T. Shu Y. Shan X. Zhang J. . (2023). The whole-genome sequencing of prevalent DENV-1 strains during the largest dengue virus outbreak in Xishuangbanna Dai autonomous prefecture in 2019. J. Med. Virol. 95, e28115. doi: 10.1002/jmv.28115 36059257

[B12] Liao Q. Shan Z. Wang M. Huang J. Xu R. Huang K. . (2017). An evaluation of asymptomatic dengue infections among blood donors during the 2014 dengue outbreak in Guangzhou, China. J. Med. Virol. 89, 2037–2040. doi: 10.1002/jmv.24883 28639699

[B13] Linnen J. M. Vinelli E. Sabino E. C. Tobler L. H. Hyland C. Lee T. H. . (2008). Dengue viremia in blood donors from Honduras, Brazil, and Australia. Transfusion 48, 1355–1362. doi: 10.1111/j.1537-2995.2008.01772.x 18503610

[B14] Ma J. Liang Y. Zhou Z. Feng X. Li F. Chen Q. . (2025). Whole genome sequence characteristics of 13 dengue virus serotype 2 strains isolated from Guangzhou in 2023. China Trop. Med. 25, 6. doi: 10.13604/j.cnki.46-1064/r.2025.01.18

[B15] Matos D. Tomashek K. M. Perez-Padilla J. Munoz-Jordan J. Hunsperger E. Horiuchi K. . (2016). Probable and possible transfusion-transmitted dengue associated with NS1 antigen-negative but RNA confirmed-positive red blood cells. Transfusion 56, 215–222. doi: 10.1111/trf.13288 26469514

[B16] Muller D. A. Choo J. J. Y. McElnea C. Duyen H. T. L. Wills B. Young P. R. (2025). Kinetics of NS1 and anti-NS1 IgG following dengue infection reveals likely early formation of immune complexes in secondary infected patients. Sci. Rep. 15, 6684. doi: 10.1038/s41598-025-91099-5 39994315 PMC11850851

[B17] Paz-Bailey G. Adams L. E. Deen J. Anderson K. B. Katzelnick L. C. (2024). Dengue. Lancet 403, 667–682. doi: 10.1001/jama.2024.21094 38280388 PMC12372472

[B18] Sabino E. C. Loureiro P. Lopes M. E. Capuani L. McClure C. Chowdhury D. . (2016). Transfusion-transmitted dengue and associated clinical symptoms during the 2012 epidemic in Brazil. J. Infect. Dis. 213, 694–702. doi: 10.1093/infdis/jiv326 26908780 PMC4747611

[B19] Schubert S. Weyrich A. S. Rowley J. W. (2014). A tour through the transcriptional landscape of platelets. Blood 124, 493–502. doi: 10.1182/blood-2014-04-512756 24904119 PMC4110657

[B20] Simon A. Y. Sutherland M. R. Pryzdial E. L. (2015). Dengue virus binding and replication by platelets. Blood 126, 378–385. doi: 10.1182/blood-2014-09-598029 25943787 PMC4826145

[B21] Stramer S. L. Hollinger F. B. Katz L. M. Kleinman S. Metzel P. S. Gregory K. R. . (2009). Emerging infectious disease agents and their potential threat to transfusion safety. Transfusion 49 Suppl 2, 1S–29S. doi: 10.1111/j.1537-2995.2009.02279.x 19686562

[B22] Stramer S. L. Lanteri M. C. Brodsky J. P. Foster G. A. Krysztof D. E. Groves J. A. . (2022). Mitigating the risk of transfusion-transmitted infections with vector-borne agents solely by means of pathogen reduction. Transfusion 62, 1388–1398. doi: 10.1111/trf.16950 35726756 PMC9541364

[B23] Stramer S. L. Linnen J. M. Carrick J. M. Foster G. A. Krysztof D. E. Zou S. . (2012). Dengue viremia in blood donors identified by RNA and detection of dengue transfusion transmission during the 2007 dengue outbreak in Puerto Rico. Transfusion 52, 1657–1666. doi: 10.1111/j.1537-2995.2012.03566.x 22339201

[B24] Sun J. Wu D. Zhou H. Zhang H. Guan D. He X. . (2016). The epidemiological characteristics and genetic diversity of dengue virus during the third largest historical outbreak of dengue in Guangdong, China, in 2014. J Infect. 72 (1), 80–90. 10.1016/j.jinf.2015.10.007 26546854

[B25] Sutherland M. R. Simon A. Y. Serrano K. Schubert P. Acker J. P. Pryzdial E. L. (2016). Dengue virus persists and replicates during storage of platelet and red blood cell units. Transfusion 56, 1129–1137. doi: 10.1111/trf.13454 26779802

[B26] World Health Organization (2025). Dengue and Severe Dengue. Available online at: https://www.who.int/news-room/fact-sheets/detail/dengue-and-severe-dengue (Accessed May 20, 2026).

[B27] Yan Y. Liu X. Lu S. Chang L. Dong J. Ji H. . (2025). Two-year surveillance of dengue, zika, and chikungunya viruses among Chinese blood donors - Guangxi and Yunnan PLADs, China 2022-2023. China CDC Wkly 7, 357–362. doi: 10.46234/ccdcw2025.058 40226220 PMC11983155

