# Supplementary material for: Molecular confirmation of transfusion-transmitted dengue virus infection following platelet transfusion in the Chinese mainland
Source: Front Cell Infect Microbiol. 2026 Sep 14;16:1920581. doi: 10.3389/fcimb.2026.1920581 (PMC13616782; doi:10.3389/fcimb.2026.1920581)
Supplement: Supplementary file 1 [file Image1.pdf]

	NC_001474.1	Donor	Recipient_1	Recipient_2	GZD01_2024	GZP01_2025	GZP02_2025	NC_001477.1
NC_001474.1								
Donor	0.926							
Recipient #1	0.926	1.000						
Recipient #2	0.926	1.000	1.000					
GZD01_2024	0.634	0.636	0.636	0.636				
GZP01_2025	0.634	0.636	0.636	0.636	0.997			
GZP02_2025	0.633	0.635	0.635	0.635	0.997	0.996		
NC_001477.1	0.655	0.653	0.653	0.653	0.886	0.887	0.887	

Supplemental Figure 1. Sequence identity matrix of the DENV E gene fragments. NC_001474.1 and NC_001477.1 are reference sequence of DENV-2 and DENV-1. Red borders show the identity between donor and recipients in the TT-DENV case. Blue border show the identity between DF (mosquito borne) individuals.
